# Brain Abscesses Complicating Bacterial Meningitis—A Nationwide Cohort Study From the Netherlands

**DOI:** 10.1111/ene.70524

**Published:** 2026-02-25

**Authors:** Fabian D. Liechti, Evelien H. G. M. Drost, Merijn W. Bijlsma, Matthijs C. Brouwer, Diederik van de Beek

**Affiliations:** ^1^ Department of Neurology, Amsterdam Neuroscience Amsterdam UMC, University of Amsterdam Amsterdam the Netherlands; ^2^ Department of General Internal Medicine, Inselspital, Bern University Hospital University of Bern Bern Switzerland; ^3^ Department of Paediatrics, Amsterdam Neuroscience Amsterdam UMC Amsterdam the Netherlands

**Keywords:** bacterial meningitis, brain abscesses, central nervous system infections, mastoiditis, pneumococcal infections

## Abstract

**Background:**

Brain abscesses rarely complicate bacterial meningitis while their clinical characteristics and outcomes have not been systematically assessed. We studied prevalence, pathogen distribution, clinical characteristics including treatment, and outcomes of brain abscess in bacterial meningitis patients.

**Methods:**

In a prospective Dutch national cohort of community‐acquired bacterial meningitis (January 2006 to October 2023), we identified patients with a brain abscess diagnosed by the treating physician. Presence of abscesses was confirmed by re‐evaluation of the cranial imaging.

**Results:**

Of the 2918 episodes included, 56 were complicated by a brain abscess (prevalence 1.9%, 95% confidence interval [CI] 1.5–2.5). For *Streptococcus pneumoniae* the prevalence of abscesses among meningitis episodes was 1.1% (95% CI 0.7%–1.6%) while it was 3.6% (95% CI 2.6%–5.0%) for other bacteria. Prevalence was highest in meningitis caused by the *Streptococcus anginosus* group (11/21, 52.4%), *Klebsiella pneumoniae* (1/10, 10.0%), *Listeria monocytogenes* (8/175, 4.6%), *Escherichia coli* (1/22, 4.5%), and group A streptococcus (3/79, 3.8%). Prevalence increased between 2006–2014 and 2015–2023, with a ratio of 1.8 (95% CI 1.7–2.1). In meningitis patients with abscesses, 14‐day survival was 94% and 40 of 56 (71%) patients had an unfavourable outcome. In 9 of 56 (16%) patients the abscess was treated with a neurosurgical intervention (extra‐ventricular CSF drain or abscess aspiration). All received prolonged antibiotic treatment.

**Conclusions:**

Most brain abscesses among meningitis patients were caused by pneumococci, but non‐pneumococcal streptococci had the highest risk for abscesses. Patients with abscesses had a high risk for an unfavourable outcome.

## Graphical abstract

Prevalence, pathogen distribution, clinical characteristics, and outcomes of brain abscess in bacterial meningitis was assessed in this study.

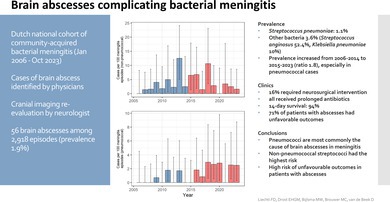

## 1 Introduction

Bacterial meningitis is a severe disease with a high risk of neurological and systemic complications [1]. Brain abscesses are an uncommon complication of bacterial meningitis [2, 3], and have been estimated to occur in 2% of community‐acquired bacterial meningitis episodes [4, 5]. However, in specific pathogens, prevalence may be higher with proportions up to 10% in meningitis due to *Listeria monocytogenes* [6, 7] or group A streptococci [8].

So far, mostly small or pathogen‐specific case series were published on bacterial meningitis patients in which the clinical course is complicated by brain abscesses [5, 9]. The prevalence of brain abscesses among meningitis patients, the pathogen distribution and the relative risk for each pathogen to cause brain abscesses may have changed, for example, following the introduction of routine vaccination with pneumococcal conjugate vaccines (PCV). Furthermore, the clinical characteristics and outcomes of meningitis patients with concomitant brain abscess have not been systematically assessed in larger case series [5]. The recently published European Society of Clinical Microbiology and Infectious Diseases guidelines contain recommendations on treatment of brain abscesses, but do not include specific treatment recommendations for the special case of community‐acquired meningitis with concomitant brain abscesses [2]. We therefore aimed to describe prevalence, pathogen distribution, and clinical characteristics including treatment practices, and outcome of patients with brain abscesses complicating bacterial meningitis in our existing prospective cohort covering almost two decades and give an update to a previously published series of 14 cases [5].

## 2 Methods

We included adult patients with community‐acquired bacterial meningitis in a nationwide prospective cohort in the Netherlands (MeninGene) between January 2006 and October 2023. Written informed consent to participate in the study was obtained from all patients or their legally authorised representatives. The cohort study was approved by the local ethical committee as previously described (approved by the Medical Ethics Committee of the Academic Medical Center, Amsterdam, the Netherlands, approval number NL43784.018.13, March 18, 2013) [10, 11]. In brief, patients 16 years of age or older were recruited by notification from the Netherlands Reference Laboratory for Bacterial Meningitis (NRLBM), which receives more than 90% of isolates of patients with bacterial meningitis in the Netherlands and performs serotyping [12]. Physicians could also include patients without report of the NRLBM [11]. All patients included had clinical signs and symptoms of meningitis and either a positive cerebrospinal fluid (CSF) culture or at least one individual CSF predictive factor for bacterial meningitis (Spanos criteria), defined as a CSF leukocyte count of > 2000 cells/mm^3^, more than 1180 polymorphonuclear leukocytes per mm^3^, CSF‐serum glucose ratio < 0.23, CSF protein > 2.2 g/L, or CSF glucose < 1.9 mmol/L [13]. Patients who did not present with typical clinical or CSF features of meningitis were not included. Episodes with recent neurosurgery, head injury or a neurosurgical device were excluded. We included cases of brain abscess secondary to bacterial meningitis based on discharge letters and radiology reports. Additionally, one author (E.D.) reviewed also cases with a subdural empyema reported for presence of an abscess. If no neuroimaging was performed, we assumed absence of brain abscess. For cases reported to be complicated by a brain abscess, cranial MRI or CT scans were reviewed for confirmation by one author (E.D.). The number of abscesses was scored as was the maximal diameter. Interventions were recorded if performed for the brain abscess and were categorised as abscess aspiration, placement of an extra‐ventricular CSF drain, decompression surgery, or none. Patients reporting alcoholism, diabetes mellitus, immunosuppressive treatment, human immunodeficiency virus, or splenectomy were categorised as immunocompromised. Underlying conditions associated with development of a brain abscess were systematically scored using the electronic case report forms, discharge letters and radiological reports and included (1) an identified or suspected primary embolic source (e.g., endocarditis); (2) a *per continuitatem* infection (mastoiditis, sinusitis, or dental focus with radiological signs of rupture to the central nervous system); or (3) meningitis complicated by brain abscess formation without other identified focus of infection [5]. For comparisons over time, the inclusion period was split in two halves, that is, January 2006 to December 2014 versus January 2015 to October 2023. Missing values are reported when applicable.

Data are displayed as mean with standard deviation (SD) or median with interquartile range (IQR) as appropriate and 95% confidence intervals (95% CI). To analyse treatment and survival, we grouped patients according to one of three pathogen categories ( *Streptococcus pneumoniae* , other Gram‐positive bacteria, Gram‐negative bacteria), excluding culture negatives. To identify indicators for a brain abscess during hospitalisation, we applied a univariate logistic regression model using registered complications (transfer to intensive care, impaired consciousness, circulatory shock, mechanical ventilation, persisting fever, pneumoniae, focal neurological deficit, seizure) and present odds ratios (OR). Unfavourable outcome (Glasgow Outcome Scale score 1–4 vs. 5) was assessed using a logistic regression model, adjusted with cubic splines for age. Analyses were done using R Statistical Software (v4.2.1; R Core Team 2021; Table S1). The study was reported according to the Strengthening the Reporting of Observational Studies in Epidemiology (STROBE) recommendations [14].

## 3 Results

**FIGURE 1:**
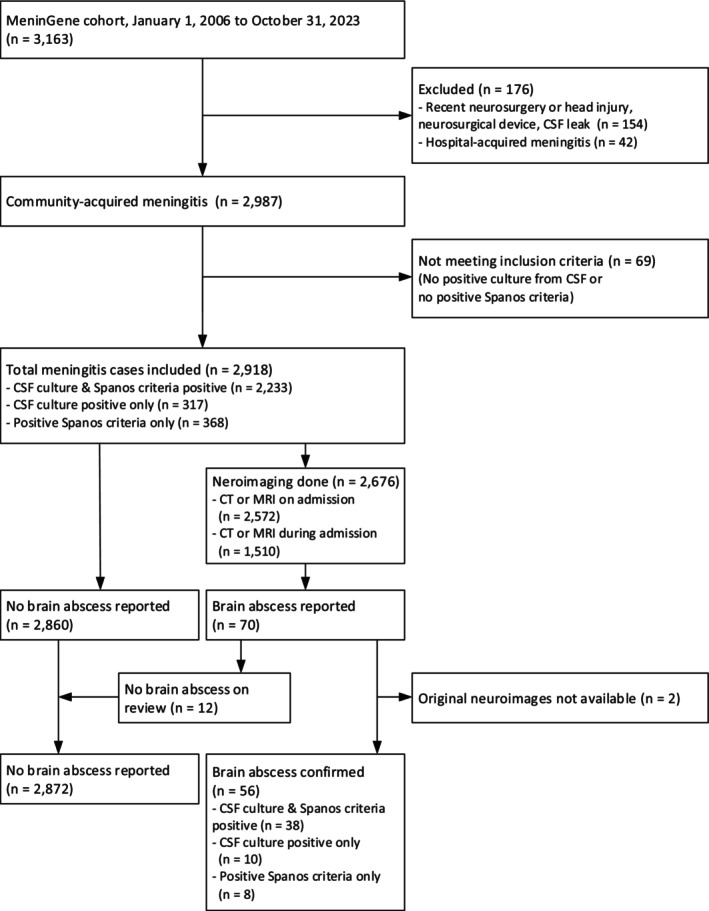
Flow‐chart. Flow‐chart of the case‐selection process.

A total of 2918 episodes of community‐acquired bacterial meningitis (Figure 1) were included, most of which were caused by *S. pneumoniae* (1956 episodes, 67%). In 56 of 2918 episodes, one or more brain abscesses were found and confirmed by the investigators (Table S2). In 14 of the 56 (25%) patients, the abscess was detected upon presentation; in all other patients, the diagnosis was made during admission. Bacterial meningitis patients with a brain abscess had a median age of 58 years (IQR 48–67 years), and 29 of the 56 patients (52%) were male (Table 1).

**TABLE 1:** Admission characteristics of patients with bacterial meningitis complicated by brain abscess.

Characteristic	Brain abscess − *N* = 56^a^
Age at presentation (years)	58 (48–67)
Male	29/56 (52%)
Immunocompromised condition^b^	19/56 (34%)
Fever on admission	39/54 (72%)
Systolic blood pressure (mmHg)^c^	145 (128–158)
Heart rate (beats per minute)^c^	88 (76–106)
Cranial nerve palsy	11/50 (22%)
Focal neurological deficit on admission	14/53 (26%)
Score on Glasgow Coma Scale^d^	13 (10–15)
Symptoms < 24 h	34/52 (65%)
Otitis or sinusitis	16/54 (30%)
Endocarditis	4/54 (7.4%)
Headache	33/45 (73%)
Blood leukocytes (per μL)^e^	15 (11–22)
Blood thrombocytes (per μL)^f^	182 (155–276)
C‐reactive protein (mg/L)^g^	191 (91–316)
Blood culture positive	31/52 (60%)
CSF leukocytes (per μL)^h^	2304 (495–6349)
CSF opening pressure (cm H_2_O)^i^	35 (27–50)

Abbreviation: CSF, cerebrospinal fluid.

^a^ Median (IQR); *n* (%).

^b^ Alcoholism, diabetes, splenectomy, immunosuppressive therapy, or HIV.

^c^ Blood pressure and heart rate was known for 52 patients.

^d^ Glasgow Coma Scale was known for all patients.

^e^ Blood leukocyte count was known for 54 patients.

^f^ Blood thrombocyte count was known for 49 patients.

^g^ C‐reactive protein level was known for 54 patients.

^h^ CSF leukocyte count was known for 55 patients.

^i^ CSF leukocyte count was known for 23 patients.

### 3.1 Epidemiology

The overall prevalence of brain abscess in patients with bacterial meningitis was 1.9% (95% CI 1.5%–2.5%). The probability of an identified abscess per meningitis episode was 3.6% (95% CI 2.6%–5.0%) for non‐pneumococcal meningitis and 1.1% (95% CI 0.7%–1.6%) for pneumococcal meningitis.

The relative risk was particularly high for episodes caused by the *S. anginosus* group (11 of 21 episodes, 52%), *L. monocytogenes* (8 of 173 episodes, 5%), and *S. pyogenes* (3 of 79 episodes, 4%). The prevalence also varied by age group (Table S3). The prevalence of brain abscesses in meningococcal meningitis (3 of 306 episodes, 1%) was similar to that observed in pneumococcal meningitis. Among the 56 brain abscess cases, the most common pathogen was *S. pneumoniae*, identified in 21 cases (38%). Other identified pathogens included the *Streptococcus anginosus* group (11 cases, 20%), *L. monocytogenes* (8 cases, 14%), *N. meningitidis* (3 cases, 5%), and *Streptococcus pyogenes* (3 cases, 5%). Those with 2 or less cases are listed in Table S4. Among the *S. pneumoniae* cases for which serotyping was available, serotype 3 was the most common, detected in 5 of 18 (28%) cases.

### 3.2 Clinical Characteristics and Ancillary Investigations

Of the 56 cases, 19 (34%) were immunocompromised. This included diabetes in 9 of 56 (16%), alcoholism in 4 of 55 (7%), and immunosuppressive therapy in 11 of 55 (20%). Additionally, 10 of 56 (18%) cases had a history of cancer.

At admission, 34 of 52 (65%) reported symptom onset within 24 h, 33 of 45 (73%) had headache, and 39 of 54 (72%) presented with fever. On neurologic examination, 14 of 53 (26%) showed focal neurological deficits. The median Glasgow Coma Scale score was 13 (IQR 10–15). Elevated blood leukocytes (> 10,000 G/L) were observed in 44 of 54 (81%) cases, normal counts in 8 (15%), and leukopenia (< 4000 G/L) in 2 (4%). The median CSF leukocyte count was 2059/mm^3^ (IQR 1019–5494/mm^3^), and the median CSF opening pressure was 37 cm H_2_O (IQR 35–50 cm H_2_O). Brain abscess was associated with cranial nerve palsy (OR 3.3, 95% CI 1.7–10.0, *p* < 0.001), endocarditis (OR 3.8, 95% CI 1.1–9.7, *p* = 0.013), and negative blood cultures (OR 2.0, 95% CI 1.1–3.3, *p* = 0.015). However, there was no association with immunosuppression, otitis–sinusitis, or headache.

Neuroimaging was done in 2676 (92%) of all bacterial meningitis episodes. It was performed on admission only in 1166 (40%), during admission only in 104 (4%), and both on and during admission in 1406 (48%) episodes. Among the 56 brain abscess cases, the median maximal abscess diameter was 17 mm (IQR 11–25 mm). A single abscess was identified in 32 of 56 (57%) patients, 2–4 abscesses in 10 of 56 (18%) patients, and more than 4 abscesses in 14 of 56 (25%) patients. Additional neuroimaging findings included signs of intraventricular rupture or ventriculitis in 15 patients (27%), subdural empyema in 11 (20%), cerebral infarction in 9 (16%), and intracranial haemorrhage in 3 (5%).

Among bacterial meningitis episodes with brain abscess, blood cultures were positive in 31 of 52 cases (60%). Meningitis was diagnosed by positive CSF culture in 48 of 56 (86%) patients and by positive Spanos criteria alone in 8 (14%).

A presumed *per continuitatem* infection was observed in 17 cases, most commonly in *S. pneumoniae* (*n* = 10, 59%), *S. pyogenes* (*n* = 3, 18%), and the *S. anginosus* group (*n* = 2, 12%). An embolic source was identified or presumed in 7 patients: *S. anginosus* group, *Staphylococcus aureus* , *Escherichia coli* , *Nocardia farcinica* , and *Aggregatibacter aphrophilus* in one case each (14%), and *S. pneumoniae* in 2 cases (29%).

No other infectious focus was identified in 32 of 56 patients (57%). Of the 32 patients with no identifiable focus, 18 (56%) exhibited multiple brain abscesses. The most commonly identified pathogens in these 32 patients were *S. pneumoniae* (28%, *n* = 9), the *S. anginosus* group (25%, *n* = 8), and *L. monocytogenes* (25%, *n* = 8).

### 3.3 Treatment, Complications, and Outcomes

In 4 of the 56 cases (9%) antibiotic treatment was started before admission. Treatment with adjunctive dexamethasone was started in 44 of 56 (79%) patients. All 50 surviving meningitis patients with brain abscess received prolonged antibiotic treatment, that is, longer than 14 days. The 7 patients, who died between day 2 and 39 after admission, were all continuously treated until death or until switch to palliative care only; none of these deaths were due to early discontinuation of antibiotic treatment followed by relapse. In cases with a *per continuitatem* infection, treatment often included local source control, consisting of mastoidectomy in 4 of 12 (33%) patients with mastoiditis, but not in the 4 cases with sinusitis. Dental surgery was performed in the one patient with a dentogen focus.

Neurosurgical intervention was performed in 12 of 56 cases (21%). In 6 patients, an external ventricular drain was inserted because of obstructive hydrocephalus; one of these patients subsequently underwent hemicraniectomy because of an extensive cerebral abscess with subdural collections. In 3 patients the abscess was managed by drainage (*n* = 2) or aspiration (*n* = 1); in 1 patient, a biopsy was performed. Additionally, burr hole evacuation was performed in 2 patients because of concurrent subdural empyema.

During hospitalisation, 12 of 54 (22%) bacterial meningitis patients with a brain abscess had persisting fever, 40 of 54 (74%) had focal neurological deficits, and 14 of 54 (26%) developed seizures as a complication (Table 2). The length of hospital stay until discharge or in‐hospital death in patients with concomitant brain abscess was 38 days (IQR 24–59d). Among the 56 patients with a brain abscess, 7 (13%) died during the hospital stay and 33 of 49 (67%) survivors were disabled (GOS 2–4) at discharge. In the age‐adjusted logistic regression model, brain abscess as a complication of bacterial meningitis was associated with unfavourable outcome (OR 4.9, 95% CI 2.7–9.3), but the in‐hospital survival probability was higher (*p* = 0.005).

**TABLE 2:** Complications and outcomes during hospitalisation in patients with bacterial meningitis and concomitant brain abscess.

Characteristic	Yes, *N* = 56^a^
Transfer to intensive care	17/56 (30%)
Impaired consciousness	30/52 (58%)
Circulatory shock	5/55 (9%)
Mechanical ventilation	19/54 (35%)
Persisting fever	12/54 (22%)
Pneumonia	9/54 (17%)
Focal neurological deficit	40/54 (74%)
Seizure	14/54 (26%)
Days to death	22 (11–31)
Length of hospital stay^b^	38 (24–59)
Glasgow Outcome Scale	
Dead	7/56 (13%)
Vegetative survival	1/56 (2%)
Severely disabled	11/56 (20%)
Moderately disabled	21/56 (38%)
Good recovery	16/56 (29%)

^a^ Median (IQR); *n* (%).

^b^ Until discharge or death.

### 3.4 Changes Over Time

Between 2006 and 2014, neuroimaging was performed in 1378 of 1529 bacterial meningitis episodes (90.1%, 95% CI 89.4%–90.9%), compared to 1298 of 1389 episodes (93.4%, 95% CI 92.8%–94.1%) between 2015 and 2023. Brain abscesses were reported in 21 of 1529 bacterial meningitis episodes (1.4%, 95% CI 0.9%–2.1%) during 2006–2014 and in 35 of 1389 episodes (2.5%, 95% CI 1.8%–3.5%) during 2015–2023, corresponding to a prevalence ratio (PR) of 1.8 (95% CI 1.7–2.1).

**FIGURE 2:**
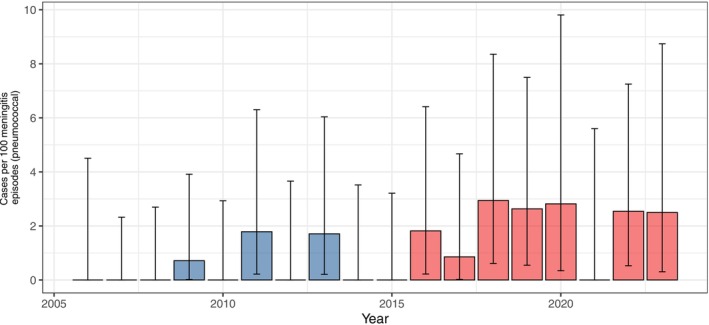
Episodes caused by *S. pneumoniae* . Annual proportion of brain abscess as complication of meningitis in the first half (2006–2014, light blue bars) and second half (2015–2023, light red bars) of the study period with 95% CI.

**FIGURE 3:**
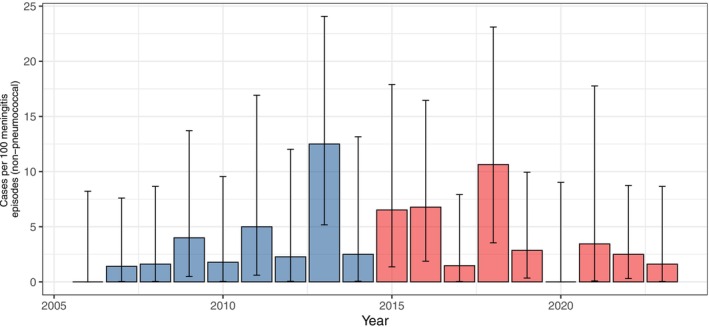
Episodes caused by non‐pneumococcal bacteria. Annual proportion of brain abscess as complication of meningitis in the first half (2006–2014, light blue bars) and second half (2015–2023, light red bars) of the study period with 95% CI.

This increase was primarily driven by episodes caused by *S. pneumoniae* , with a prevalence of abscesses among pneumococcal meningitis episodes rising from 0.5 (95% CI 0.2–1.1) in 2006–2014 to 1.8 (95% CI 1.0–2.9) in 2015–2023 (PR 3.8, 95% CI 2.7–6.8; Figures 2 and 3). Notably, this trend occurred despite a slight decrease in the overall proportion of pneumococcal meningitis, from 70% (1067 of 1529 episodes) in 2006–2014 to 64% (889 of 1389 episodes) in 2015–2023. Serotype 3 was documented in 2 brain abscess cases in 2006–2014 and in 3 cases in 2015–2023 (PR 1.3, 95% CI 1.1–2.3). For other bacteria ( *S. anginosus* group, *L. monocytogenes* , *S. pyogenes* ), no increase in brain abscess incidence was observed during the study period (PR 1.1, 95% CI 0.9–2.3, PR 0.9, 95% CI 0.9–0.9, and PR 0.9, 95% CI 0.6–4.5, respectively).

## 4 Discussion

We observed an increasing prevalence of brain abscesses since 2015, particularly among pneumococcal meningitis episodes. We did not find any signs or symptoms that could predict the presence of a concomitant brain abscess. Therefore, a brain abscess should be in the differential diagnosis in bacterial meningitis who fail to improve or worsen during admission. Neuroimaging, preferably MRI, should be performed in these patients and neurosurgical treatment may be indicated when an abscess is identified.

In about half of the patients, an embolic source of infection or *per continuitatem* infection was identified. As such infections may require additional treatment such as cardiac or ENT surgery, all meningitis patients with a brain abscess should be analysed by a cardiologist with cardiac ultrasound and an ENT specialist for concomitant sinusitis, otitis, or mastoiditis. Specifically, in meningitis caused by *S. aureus* , *E. coli* , or *A. aphrophilus* , a central embolic source was frequently identified. In a large proportion of patients, no underlying focus of infection was identified, but we were unable to ascertain whether the proper work‐up was done in all of these patients. A notable characteristic of these patients was that there were often multiple brain abscesses, suggesting hematogenic spread. This was relatively frequently observed among cases caused by *S. pneumoniae* , *L. monocytogenes* , or *N. meningitidis* .

The increasing prevalence over time may be a result of an increased number or higher sensitivity of reporting, including better performance or higher number of neuroimaging; although if this were true a similar increase in non‐pneumococcal meningitis would have been expected. The increasing prevalence of brain abscesses among pneumococcal meningitis may also be due to changes in genotype or serotype distributions facilitating development of abscesses in infected persons. Pneumococcal serotype 3 has repeatedly been associated with complicating invasive disease, for example, causing abscesses in paediatric pneumonia [15, 16]. However, as the number of cases caused by individual pneumococcal serotypes is low, statistical differences in serotype distribution are difficult to identify.

Nine pathogens posed an increased risk for brain abscesses in patients with bacterial meningitis. In meningitis caused by Group C streptococci, *A. aphrophilus* , *Nocardia farcinica* , the *S. anginosus* group, and *H. parainfluenzae* 50% or more of patients had a brain abscesses. In meningitis caused by *L. monocytogenes* , *S. pyogenes* (Group A streptococci), *K. pneumoniae* , *E. coli* , *S. aureus* , and those with negative bacterial cultures from CSF and blood, the proportion of those with concomitant brain abscess was between 2% and 10%. Together, in 438 of 2288 (5%) meningitis patients one of these pathogens was identified. In these cases, the threshold to perform neuroimaging should be very low. Patients who had a focal neurological deficit or seizures during hospitalisation and those with persisting fever were at high risk for finding brain abscesses on neuroimaging. Such symptoms should thus prompt performance of neuroimaging, preferably MRI, given that presence of an abscess will impact further treatment [2]. All patients received prolonged intravenous antibiotic treatment, in line with the European Society of Clinical Microbiology and Infectious Diseases guidelines for the diagnosis and treatment of brain abscesses, which recommend 6–8 weeks of intravenous antimicrobial treatment. Abscess aspiration or drainage was rarely performed in the current cohort, but should be discussed on case‐by‐case basis to improve clearance. In meningitis patients with concomitant abscess, antibiotic treatment should not be withheld or delayed, as the pathogen can be identified in the CSF and immediate antibiotic treatment is warranted, in contrast to primary brain abscesses where it is recommended to aspirate the abscess first to identify the pathogen [2].

Strength of our analysis is the heterogeneous sample from a prospective, nationwide cohort study, covering almost 20 years, and enabling comparison with controls who also had bacterial meningitis. Limitations to this study are, first, the still limited sample size for many pathogens. However, this is currently the largest cohort on bacterial meningitis patients complicated by brain abscesses reported—a population that is diverse from those presenting with primary brain abscess. Second, according to our inclusion criteria, we did not differentiate between primary and secondary brain abscesses, such as those resulting from intraventricular rupture of an abscess. Nevertheless, the cases presented in this study reflect the clinical reality of meningitis patients eventually diagnosed with concomitant brain abscess during hospitalisation. Finally, cranial imaging in this cohort study was not performed according to a specific protocol and timing of imaging differed between patients. Some brain abscesses may have been missed in initial CT evaluation when brain abscesses were small, and we did not systematically follow up patients for readmission due to later detected brain abscesses missed during the initial admission.

## 5 Conclusions

*S. pneumoniae* is the most common causative pathogen of brain abscesses complicating community‐acquired bacterial meningitis, but the individual risk of a patient with pneumococcal meningitis developing an abscess is relatively low. In contrast, the relative risk is higher for patients with meningitis caused by Group C streptococci, *A. aphrophilus* , *Nocardia farcinica* , the *S. anginosus* group, or *H. parainfluenzae* (all risk ≥ 50%), *L. monocytogenes* , *S. pyogenes* (group A streptococci), *K. pneumoniae* , *E. coli* , or *S. aureus* (2%–10%). We found an increased risk of abscess in *S. pneumoniae* meningitis over time, which could be attributed to host or pathogen factors.

## Author Contributions

**Fabian D. Liechti:** conceptualization, methodology, software, data curation, investigation, validation, formal analysis, writing – original draft. **Evelien H. G. M. Drost:** data curation, formal analysis, writing – review and editing. **Merijn W. Bijlsma:** methodology, validation, formal analysis, supervision, writing – review and editing. **Matthijs C. Brouwer:** conceptualization, methodology, supervision, funding acquisition, project administration, resources, writing – review and editing. **Diederik van de Beek:** conceptualization, methodology, supervision, funding acquisition, visualisation, project administration, resources, writing – review and editing.

## Funding

This work was supported by the Swiss National Science Foundation (grant number P500PM_206639 to F.D.L.); the Insel Gruppe Young Investigator Award (no grant number to F.D.L.); the Netherlands Organization for Health Research and Development (ZonMw; NWO‐Vidi grant [grant number 917.17.308 to M.C.B.], NWO‐Vici grant [grant number 918.19.627 to D.v.d.B.]); the Academic Medical Centre (AMC Fellowship to D.v.d.B.); the European Research Council (ERC Consolidator grant to M.C.B. [grant number 101011237 to M.C.B.]). The funders had no role in study design, data collection and analysis, decision to publish, or preparation of the manuscript.

## Disclosure

The authors have nothing to report.

## Conflicts of Interest

The authors declare no conflicts of interest.

## Supporting information


**Table S1:** List of R packages used.
**Table S2:** Cases of bacterial meningitis with concomitant brain abscess.
**Table S3:** Pathogens identified in patients with brain abscess, stratified by the age group.
**Table S4:** Proportions of brain abscess among patients with bacterial meningitis, stratified by pathogen identified.

## Data Availability

Although data protection regulations in the Netherlands do not allow sharing of individual participant data, datasets with selected aggregated data are available upon reasonable request. Individuals who request data will be asked to sign a data access agreement.
