# Supplementary material for: Brain Abscesses Complicating Bacterial Meningitis—A Nationwide Cohort Study From the Netherlands
Source: Eur J Neurol. 2026 Feb 25;33(2):e70524. doi: 10.1111/ene.70524 (PMC12933248; doi:10.1111/ene.70524)
Supplement: Supplementary file 1 — Table S1: List of R packages used. Table S2: Cases of bacterial meningitis with concomitant brain abscess. Table S3: Pathogens identified in patients with brain abscess, stratified by the age group. Table S4: Proportions of brain abscess among patients with bacterial meningitis, stratified by pathogen identified. [file ENE-33-e70524-s001.docx]

# Supplement

[**Supplementary table 1.** List of R packages used. 2](#_Toc207111730)

[**Supplementary table 2.** Cases of bacterial meningitis with concomitant brain abscess. 3](#_Toc207111731)

[**Supplementary table 3.** Pathogens identified in patients with brain abscess, stratified by the age group. 6](#_Toc207111732)

[**Supplementary table 4.**  Proportions of brain abscess among patients with bacterial meningitis, stratified by pathogen identified. 7](#_Toc207111733)

## Supplementary tables

**Supplementary table 1.** List of R packages used.

| - Aragon T (2020). epitools: Epidemiology Tools. R package version 0.5-10.1, <https://CRAN.R-project.org/package=epitools>  - Grolemund G, Wickham H (2011). “Dates and Times Made Easy with lubridate.” Journal of Statistical Software, *40*(3), 1-25. <https://www.jstatsoft.org/v40/i03/>  - Müller K, Wickham H (2023). tibble: Simple Data Frames. R package version 3.2.1, <https://CRAN.R-project.org/package=tibble>  - R Core Team (2022). R: A Language and Environment for Statistical Computing. R Foundation for Statistical Computing, Vienna, Austria. <https://www.R-project.org/>  - Rudis B, Bolker B, Schulz J (2017). ggalt: Extra Coordinate Systems, 'Geoms', Statistical Transformations, Scales and Fonts for 'ggplot2'. R package version 0.4.0, <https://CRAN.R-project.org/package=ggalt>  - Sjoberg D, Whiting K, Curry M, Lavery J, Larmarange J (2021). “Reproducible Summary Tables with the gtsummary Package.” The R Journal, *13*, 570-580. doi:10.32614/RJ-2021-053 <https://doi.org/10.32614/RJ-2021-053>  - Therneau T (2022). A Package for Survival Analysis in R. R package version 3.3-1, <https://CRAN.R-project.org/package=survival>. Terry M. Therneau, Patricia M. Grambsch (2000). Modeling Survival Data: Extending the Cox Model. Springer, New York. ISBN 0-387-98784-3.  - Wickham H (2016). ggplot2: Elegant Graphics for Data Analysis. Springer-Verlag New York. ISBN 978-3-319-24277-4, <https://ggplot2.tidyverse.org>  - Wickham H (2023). forcats: Tools for Working with Categorical Variables (Factors). R package version 1.0.0, <https://CRAN.R-project.org/package=forcats>  - Wickham H (2023). stringr: Simple, Consistent Wrappers for Common String Operations. R package version 1.5.1, <https://CRAN.R-project.org/package=stringr>  - Wickham H, Averick M, Bryan J, Chang W, McGowan LD, François R, Grolemund G, Hayes A, Henry L, Hester J, Kuhn M, Pedersen TL, Miller E, Bache SM, Müller K, Ooms J, Robinson D, Seidel DP, Spinu V, Takahashi K, Vaughan D, Wilke C, Woo K, Yutani H (2019). “Welcome to the tidyverse.” Journal of Open Source Software, *4*(43), 1686. doi:10.21105/joss.01686 <https://doi.org/10.21105/joss.01686>  - Wickham H, François R, Henry L, Müller K, Vaughan D (2023). dplyr: A Grammar of Data Manipulation. R package version 1.1.4, <https://CRAN.R-project.org/package=dplyr>  - Wickham H, Henry L (2023). purrr: Functional Programming Tools. R package version 1.0.2, <https://CRAN.R-project.org/package=purrr>  - Wickham H, Hester J, Bryan J (2024). readr: Read Rectangular Text Data. R package version 2.1.5, <https://CRAN.R-project.org/package=readr>  - Wickham H, Vaughan D, Girlich M (2023). tidyr: Tidy Messy Data. R package version 1.3.0, <https://CRAN.R-project.org/package=tidyr> |
| --- |

**Supplementary table 2.** Cases of bacterial meningitis with concomitant brain abscess.

| Age group | Otitis or sinusitis | Indicative CSF (Spanos criteria) | CSF culture | Blood culture | Pathogen | Brain abscess present on admission | Category | Multiple abscesses | Intervention |
| --- | --- | --- | --- | --- | --- | --- | --- | --- | --- |
| Younger adults (16–44 years) | No | Yes | Pos. | Pos. | Group C streptococcus | absent | Cerebritis | Yes | No |
| Younger adults (16–44 years) | Yes | Yes | Neg. | Pos. | Group C streptococcus | absent | Per continuitatem | No | No |
| Older adults (>65 years) | No | Yes | Pos. | Neg. | *L. monocytogenes* | absent | Cerebritis | Yes | No |
| Younger adults (16–44 years) | No | Yes | Pos. | Neg. | *L. monocytogenes* | present | Cerebritis | Yes | No |
| Adults (45–64 years) | No | No | Pos. | Pos. | *L. monocytogenes* | present | Cerebritis | No | No |
| Older adults (>65 years) | No | No | Pos. | Pos. | *L. monocytogenes* | absent | Cerebritis | Yes | No |
| Older adults (>65 years) | No | Yes | Pos. | Pos. | *L. monocytogenes* | present | Cerebritis | No | No |
| Older adults (>65 years) | No | No | Pos. | Pos. | *L. monocytogenes* | present | Cerebritis | Yes | No |
| Adults (45–64 years) | No | Yes | Pos. | Neg. | *L. monocytogenes* | present | Cerebritis | Yes | No |
| Older adults (>65 years) | No | Yes | Pos. | Neg. | *L. monocytogenes* | present | Cerebritis | Yes | No |
| Adults (45–64 years) | No | Yes | Pos. | Neg. | *Nocardia farcinica* | absent | Embolic | Yes | No |
| Older adults (>65 years) | No | Yes | Pos. | Neg. | *S. anginosus group* | present | Cerebritis | No | EVD |
| Adults (45–64 years) | No | Yes | Pos. | Neg. | *S. anginosus group* | absent | Embolic | Yes | EVD |
| Younger adults (16–44 years) | No | Yes | Pos. | Pos. | *S. anginosus group* | absent | Cerebritis | No | No |
| Adults (45–64 years) | No | Yes | Neg. | Neg. | *S. anginosus group* | present | Cerebritis | No | No |
| Adults (45–64 years) | No | Yes | Pos. | Neg. | *S. anginosus group* | absent | Cerebritis | Yes | Biopsy |
| Younger adults (16–44 years) | Yes | Yes | Pos. | Neg. | *S. anginosus group* | absent | Per continuitatem | No | EVD, decompression surgery |
| Younger adults (16–44 years) | Yes | Yes | Pos. | Neg. | *S. anginosus group* | absent | Per continuitatem | No | EVD |
| Adults (45–64 years) | No | Yes | Pos. | Pos. | *S. anginosus group* | NA | Cerebritis | No | EVD |
| Adults (45–64 years) | No | Yes | Neg. | Pos. | *S. anginosus group* | absent | Cerebritis | No | No |
| Adults (45–64 years) | No | Yes | Neg. | Pos. | *S. anginosus group* | absent | Cerebritis | Yes | No |
| Younger adults (16–44 years) | No | No | Pos. | Neg. | *S. anginosus group* | present | Cerebritis | No | No |
| Adults (45–64 years) | No | No | Pos. | Pos. | *S. aureus* | absent | Embolic | Yes | EVD |
| Adults (45–64 years) | No | Yes | Pos. | Pos. | *S. pneumoniae* | absent | Cerebritis | Yes | No |
| Older adults (>65 years) | No | Yes | Pos. | Neg. | *S. pneumoniae* | absent | Cerebritis | Yes | No |
| Older adults (>65 years) | No | Yes | Pos. | Neg. | *S. pneumoniae* | absent | Cerebritis | Yes | No |
| Younger adults (16–44 years) | Yes | No | Pos. | Pos. | *S. pneumoniae* | NA | Per continuitatem | No | No |
| Older adults (>65 years) | No | Yes | Pos. | Pos. | *S. pneumoniae* | NA | Cerebritis | Yes | No |
| Adults (45–64 years) | Yes | Yes | Pos. | Pos. | *S. pneumoniae* | present | Per continuitatem | No | Drainage |
| Older adults (>65 years) | No | NA | Pos. | Pos. | *S. pneumoniae* | absent | Embolic | Yes | No |
| Older adults (>65 years) | No | Yes | Pos. | Pos. | *S. pneumoniae* | absent | Cerebritis | No | No |
| Adults (45–64 years) | Yes | Yes | Pos. | Neg. | *S. pneumoniae* | absent | Per continuitatem | No | No |
| Adults (45–64 years) | NA | Yes | Pos. | Pos. | *S. pneumoniae* | absent | Per continuitatem | Yes | No |
| Older adults (>65 years) | No | Yes | Pos. | NA | *S. pneumoniae* | absent | Cerebritis | No | No |
| Adults (45–64 years) | No | Yes | Pos. | NA | *S. pneumoniae* | absent | Cerebritis | Yes | No |
| Adults (45–64 years) | No | Yes | Pos. | Pos. | *S. pneumoniae* | absent | Embolic | No | No |
| Adults (45–64 years) | Yes | Yes | Pos. | Pos. | *S. pneumoniae* | absent | Per continuitatem | No | No |
| Adults (45–64 years) | Yes | Yes | Pos. | Pos. | *S. pneumoniae* | absent | Per continuitatem | No | No |
| Adults (45–64 years) | No | Yes | Pos. | Pos. | *S. pneumoniae* | absent | Cerebritis | No | No |
| Older adults (>65 years) | Yes | Yes | Pos. | Pos. | *S. pneumoniae* | absent | Per continuitatem | No | No |
| Adults (45–64 years) | Yes | Yes | Pos. | Pos. | *S. pneumoniae* | absent | Per continuitatem | No | No |
| Adults (45–64 years) | No | Yes | Neg. | Neg. | *S. pneumoniae* | present | Cerebritis | Yes | No |
| Adults (45–64 years) | Yes | Yes | Pos. | Pos. | *S. pneumoniae* | absent | Per continuitatem | No | No |
| Older adults (>65 years) | Yes | Yes | Neg. | Pos. | *S. pneumoniae* | NA | Per continuitatem | No | No |
| Adults (45–64 years) | Yes | Yes | Pos. | Pos. | *S. pyogenes* or GAS | absent | Per continuitatem | No | No |
| Adults (45–64 years) | Yes | Yes | Pos. | Neg. | *S. pyogenes* or GAS | absent | Per continuitatem | No | No |
| Adults (45–64 years) | Yes | Yes | Pos. | Pos. | *S. pyogenes* or GAS | absent | Per continuitatem | No | No |
| Older adults (>65 years) | No | Yes | Pos. | Neg. | *A. aphrophilus* | present | Embolic | No | No |
| Older adults (>65 years) | No | No | Pos. | Pos. | *E. coli* | absent | Embolic | Yes | No |
| Adults (45–64 years) | No | Yes | Pos. | Neg. | *H. parainfluenzae* | present | Cerebritis | Yes | Drainage |
| Younger adults (16–44 years) | Yes | No | Pos. | NA | *K. pneumoniae* | absent | Per continuitatem | No | No |
| Adults (45–64 years) | No | No | Pos. | Pos. | *N. meningitidis* | NA | Cerebritis | No | No |
| Younger adults (16–44 years) | NA | Yes | Pos. | Pos. | *N. meningitidis* | absent | Cerebritis | Yes | No |
| Younger adults (16–44 years) | No | Yes | Pos. | Neg. | *N. meningitidis* | absent | Cerebritis | Yes | No |
| Adults (45–64 years) | No | Yes | Neg. | Neg. | Culture neg. | present | Cerebritis | No | No |
| Older adults (>65 years) | No | Yes | Neg. | NA | Culture neg. | absent | Cerebritis | No | No |

**Supplementary table 3.** Pathogens identified in patients with brain abscess, stratified by the age group.

| **Causative pathogen** | **Overall, N = 56** | **Younger adults (16–44 years), N = 11** | **Adults (45–64 years), N = 28** | **Older adults (>65 years), N = 17** |
| --- | --- | --- | --- | --- |
| *S. pneumoniae* | 21 (38%) | 1 (9.1%) | 12 (43%) | 8 (47%) |
| *S. anginosus* group | 11 (20%) | 4 (36%) | 6 (21%) | 1 (5.9%) |
| *L. monocytogenes* | 8 (14%) | 1 (9.1%) | 2 (7.1%) | 5 (29%) |
| *N. meningitidis* | 3 (5.4%) | 2 (18%) | 1 (3.6%) | 0 (0%) |
| *S. pyogenes* or GAS | 3 (5.4%) | 0 (0%) | 3 (11%) | 0 (0%) |
| Group C streptococcus | 2 (3.6%) | 2 (18%) | 0 (0%) | 0 (0%) |
| *A. aphrophilus* | 1 (1.8%) | 0 (0%) | 0 (0%) | 1 (5.9%) |
| *E. coli* | 1 (1.8%) | 0 (0%) | 0 (0%) | 1 (5.9%) |
| *H. parainfluenzae* | 1 (1.8%) | 0 (0%) | 1 (3.6%) | 0 (0%) |
| *K. pneumoniae* | 1 (1.8%) | 1 (9.1%) | 0 (0%) | 0 (0%) |
| *Nocardia farcinica* | 1 (1.8%) | 0 (0%) | 1 (3.6%) | 0 (0%) |
| *S. aureus* | 1 (1.8%) | 0 (0%) | 1 (3.6%) | 0 (0%) |
| Culture negative | 2 (3.6%) | 0 (0%) | 1 (3.6%) | 1 (5.9%) |

**Supplementary table 4.**  Proportions of brain abscess among patients with bacterial meningitis, stratified by pathogen identified.

|  | Controls, N = 2,232 | Cases, N = 56 | Proportion (95% CI) [%] |
| --- | --- | --- | --- |
| *S. pneumoniae* | 1917 | 21 | 1.1 (0.7–1.7) |
| *S. anginosus group* | 10 | 11 | 52.4 (32.4–71.7) |
| *L. monocytogenes* | 165 | 8 | 4.6 (2.4–8.9) |
| *S. pyogenes* or GAS | 76 | 3 | 3.8 (1.3–10.6) |
| *N. meningitidis* | 309 | 3 | 1.0 (0.3–2.8) |
| Group C streptococcus | 0 | 2 | 100 (34.2–100) |
| *A. aphrophilus* | 0 | 1 | 100 (20.7–100) |
| *Nocardia farcinica* | 0 | 1 | 100 (20.7–100) |
| *H. parainfluenzae* | 1 | 1 | 50.0 (9.5–90.5) |
| *K. pneumoniae* | 9 | 1 | 10.0 (1.8–40.4) |
| *E. coli* | 21 | 1 | 4.5 (0.8–21.8) |
| *S. aureus* | 46 | 1 | 2.1 (0.4–11.1) |
| No pathogen detected | 68 | 2 | 2.9 (0.8–9.8) |
| Other pathogens | 210 | 0 | N/A |
